# Single and Repeated Applications of Cerium Oxide Nanoparticles Differently Affect the Growth and Biomass Accumulation of *Silene flos-cuculi* L. (*Caryophyllaceae*)

**DOI:** 10.3390/nano11010229

**Published:** 2021-01-16

**Authors:** Daniel Lizzi, Alessandro Mattiello, Barbara Piani, Emanuele Gava, Guido Fellet, Luca Marchiol

**Affiliations:** 1 Department of AgriFood, Environmental and Animal Science, University of Udine, via delle Scienze 206, 33100 Udine, Italy; lizzi.daniel.1@spes.uniud.it (D.L.); alessandro.mattiello@uniud.it (A.M.); barbara.piani@uniud.it (B.P.); guido.fellet@uniud.it (G.F.); 2 Department of Life Sciences, University of Trieste, Via Licio Giorgieri 10, 34127 Trieste, Italy; 3 Laboratory of Inorganic Micro-Pollutants Regional Environmental Protection Agency of Friuli Venezia Giulia (ARPA-FVG), Via Colugna 42, 33100 Udine, Italy; emanuele.gava@arpa.fvg.it

**Keywords:** cerium oxide nanoparticles, terrestrial ecosystem, wild plant species, plant growth

## Abstract

Cerium oxide nanoparticles (*n*CeO_2_) have a wide variety of applications in industry. Models demonstrated that *n*CeO_2_ can reach environmental compartments. Studies regarding the relationships between plants and *n*CeO_2_ considered only crop species, whereas a relevant knowledge gap exists regarding wild plant species. Specimens of *Silene flos-cuculi* (*Caryophyllaceae*) were grown in greenhouse conditions in a substrate amended with a single dose (D1) and two and three doses (D2 and D3) of 20 mg kg^−1^ and 200 mg kg^−1^ *n*CeO_2_ suspensions, respectively. sp-ICP-MS and ICP-MS data demonstrated that *n*CeO_2_ was taken up by plant roots and translocated towards aerial plant fractions. Biometric variables showed that plants responded negatively to the treatments with a shortage in biomass of roots and stems. Although not at relevant concentrations, Ce was accumulated mainly in roots and plant leaves.

## 1. Introduction

Nanoscience and nanotechnology are rapidly developing in different applications, having the potential to considerably improve human life. Much progress has been made in applying the application of engineered nanomaterials (ENMs) and nano-enabled products in medicine, energy, electronics, innovative materials and many more fields [1]. On the other hand, the increase in the industrial production of ENMs inevitably leads to their release into the environment [2].

Once released in terrestrial ecosystems, ENMs enter watercourses and soils reaching the biota [3]. Since 2006, the Organization for Economic Cooperation and Development (OECD) has developed toxicity test guidelines for ENMs [4]. Currently, the endpoints/targets of such tests are the green algae *Raphidocelis subcapitata*, the daphnid *Daphnia magna*, the fish *Danio rerio*, the sediment organism *Lumbriculus variegatus*, soil microflora and terrestrial invertebrates *Enchytraeus crypticus* and *Eisenia fetida* [5].

The global biomass on Earth is dominated by plants, which are the primary producers in terrestrial and water ecosystems and represent about 80% of the biota [6]. Their life cycle is strongly dependent on their relationships with air, soil, and water. However, for that very reason, they constitute the first biological target of ENMs, and are not considered among the environmental targets of ENMs by the OECD guidelines. It would be advisable to evaluate the impact of ENMs and consider the consequences concerning the ecosystem services that plants provide [7].

The literature lacks systematic knowledge regarding the effects of ENMs on vascular plants. In part, this is due to the very high pace of research and development on nanomaterials. However, the most important reason concerns the fact that the discussion regarding the most appropriate experimental strategies is still open [8,9]. The studies carried out on crops [10,11,12] have been far more numerous compared to those on spontaneous plant species, and between the latter the papers on aquatic species largely prevail over those on terrestrial plants. According to the last “State of the World’s Plants and Fungi” release [13], the number of vascular plants species currently known is about 391,000. Only about 150 species have a significant commercial value, and 20% of them account for more than half of the plants eaten by humans [14,15]. Therefore, we optimistically assume that the relationships between ENMs and vascular plants have been studied much less than 0.05% of higher plant species, so far. Practical gap-filling actions are expected on this issue in the next future.

Cerium oxide nanoparticles (*n*CeO_2_) are a rare earth nanomaterial with several engineering and biological applications due to their catalytic, electrochemical, and optical properties [16]. With an estimated annual global production of 100–1000 tons per year, *n*CeO_2_ is among the most widely utilized metal oxide nanoparticle in Europe [17]. The Organization for Economic Cooperation and Development included *n*CeO_2_ in the list of ENMs for immediate priority testing [18].

As previously mentioned, the existing body of literature regarding the relationships between ENMs and plants is mostly focused on agricultural plant species. While this is justified concerning the potential risks of human exposure to nanomaterials through food consumption, in a broader ecological context, the impacts of ENMs on the whole primary producers should not be underrated in a broader ecological context. From this perspective, more aquatic [19,20,21] and wetland species [22,23,24] have been studied than terrestrial varieties. Concerning terrestrial ecosystems, to the best of our knowledge, *Pinus sylvestris* L. and *Quercus robur* L. are the only non-food terrestrial plant species to have been investigated for the exposure to ENMs [25].

The fate and effects of ENMs in the soil-plant system are always studied by supplying plants with ENMs at different concentrations, sizes, and shapes, and structured with several capping molecules in a single dose and at given time [26]. What remains is whether and how ENMs affect plant metabolism and plant growth under realistic conditions. Regardless of the ENMs source, plants are likely exposed to ENMs over a much longer time, at relatively lower concentrations but repeated pulses of ENMs; it is this last aspect about which we developed our experiment. The main goal of this study was to evaluate and compare the effects of a single dose and two and three repeated applications of *n*CeO_2_ at different concentrations on the growth of *Silene flos-cuculi* (L.).

## 2. Materials and Methods

### 2.1. nCeO_2_ Characterization

Nanoparticle characterization was carried out at the laboratories of the National Research Council—Institute of Science and Technology for Ceramics (Faenza, Italy). The cerium oxide nanopowders with an average particle size of 25 nm were purchased from Sigma-Aldrich (St. Louis, MO, USA). The *n*CeO_2_ had a mass weight of 172.11 g mol^−1^, density of 7.13 g mL^−1^ at 25 °C, and 99.95% purity. The size and average shape were measured with a transmission electron microscope (TEM, FEI Tecnai F20, FEI Company, Eindhoven, The Netherlands). The *n*CeO_2_ was suspended in deionized water and sonicated in a water bath for 30 min with a sonication intensity of 180 watts. The suspensions were characterized for Z—average size at pH 7 and hydrodynamic diameter (Hd), whose distributions were measured by dynamic light scattering (DLS) on a Zetasizer Nano ZS (Malvern Ltd., Worcestershire, UK).

### 2.2. Plant Material

*Silene flos-cuculi* L. (synonym *Lychnis flos-cuculi*) is a diploid polycarpic herbaceous perennial wetland plant, belonging to the *Caryophyllaceae* family. This species is native and distributed throughout Europe, where it is found in moist open habitats, along roads and flood plain, in wet meadows and pastures, but it also grows in the northern United States and eastern Canada [27]. In arable landscapes, it has become scarce because of the loss of habitats, but is still found in secondary habitats such as ditches and stream verges. The species is predominantly outcrossing, but capable of self-fertilization [28]. *S. flos-cuculi* forms vegetative rosettes with numerous flower stems that could be from 30 to 90 cm tall. The stems have barbed hairs that make the plant rough to the touch; stems grow over the foliage and end with pink flowers, which open between April and June, and many types of insects are attracted by the flower’s nectar. Another characteristic of these flowers is that they have five narrow petals divided into four lobes. The leaves are paired: the lower ones are stalked and the upper leaves present pointed apexes. The fruits consist of small capsules, containing many dark seeds, which can be dispersed mechanically [29].

### 2.3. Experimental Setup

**Figure 1:**
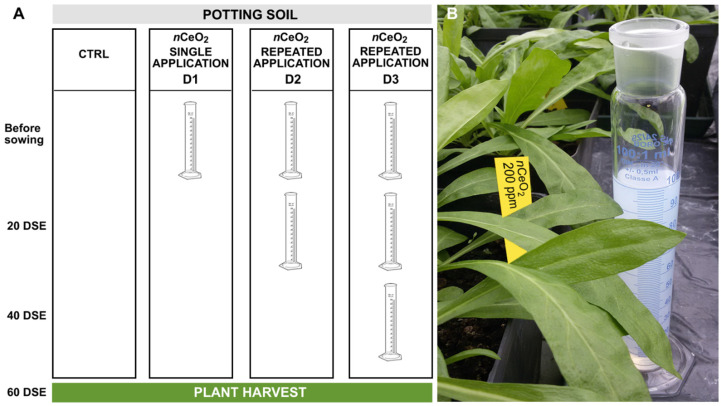
(**A**) Experimental setup showing the combination of treatments: control, single-, double-, and triple-dosed plants (Ctrl, D1, D2, and D3, respectively); (**B**) *S. flos-cuculi* plants at 40 DSE.

Seeds of *S. flos-cuculi* were purchased from SemeNostrum (Udine, Italy). The soil used for this experiment was Compo Sana organic potting mix containing forest products, compost, perlite, and fertilizer (soil pH = 6.8–7.2). The potting substrate, having a Ce concentration of 17.3 mg kg^−1^, was amended with water suspensions of *n*CeO_2_ of 25 nm in order to reach a final substrate concentration of 20 and 200 mg kg^−1^ *n*CeO_2_. Tap water was used as the control. Before sowing, *n*CeO_2_ suspensions were stirred and sonicated for 30 min to avoid the aggregation, and the first addition of *n*CeO_2_ to the substrate occurred in one dose through irrigation. The amended substrate was carefully mixed in order to obtain the prearranged concentrations. The *n*CeO_2_ amended substrates were stored in the dark at 15 °C for three days for conditioning before planting seeds. After soil equilibration, the pots were filled with 500 g soil. Repeated applications of *n*CeO_2_ were performed after 20 and 40 days from seedling emergence (DSE) in separate sets of pots. The experimental setup is showed in Figure 1. More precisely, D1 refers to the pots that received only the *n*CeO_2_ dose before sowing; D2 refers to the pots that received a second adjustment 20 DSE; and D3 refers to the pots that underwent three applications (the last one occurred 40 DSE). The additional treatments were carried out via irrigation with the solutions containing the same initial *n*CeO_2_ concentration (20 or 200 mg L^−1^). This was to attempt to recreate a situation of chronic “contamination”. The experiment was carried out in a semi-controlled greenhouse facility at the experimental farm of the University of Udine (Italy).

The trial was set up in a randomized experimental design, focusing the attention in particular on repeated treatments. Each treatment was replicated four times. Seeds were put about 2.5 cm deep in the soil and pots were placed in full sunlight, at 18–27 °C (night/day) with a relative humidity of around 60%. Two weeks after seed planting, the seedlings were thinned to two seedlings per pot. During the trial, pots were irrigated every three days and randomly reallocated every week. After 60 days, control and treated plants were harvested. Fresh plant biomass was separated into roots, stems, and leaves, and then weighed. Then, the plant fractions were thoroughly washed in tap water and rinsed three times with distilled water. In addition, roots were washed in 400 mL of 0.01 M of nitric acid in a shaker bath at 300 rpm for 5 min to remove metal ions adsorbed at the surface. Leaf area was measured using an LI-3100C Area Meter (Li-Cor Corporation, Lincoln, NE, USA). After that, the plant fractions were oven dried at 60 °C for three days, and weighed.

### 2.4. nCeO_2_ Extraction from Plant Tissues

In our study, plants grew for the entire life cycle in a solid substrate enriched with *n*CeO_2_ at the beginning and with additional treatments during the experiment. From a sub-set of pots prepared for this purpose, 20 days after the appearance of the cotyledon leaves of *S. flos-cuculi*, the plants were harvested in order to verify the entry of *n*CeO_2_ in their tissues by enzymatic digestion and single particle inductively coupled plasma mass spectrometry (spICP-MS) analysis. The plant fractions (roots, stems and leaves) were separated and in turn sent for preparation. The digesting enzyme used was Macerozyme R-10 Pectinase from *Rhizopus* sp. (Sigma-Aldrich). Small sections (0.03 g) of fresh roots, stems, and leaves were harvested, rinsed three times with deionized water, and homogenized with 8 mL of 2 mM citrate buffer at pH 4.5, using an ultrasonic bath for 5 min. After the homogenization, for every sample 2 mL of the enzyme solution (0.05 g of enzyme dissolved in 2 mL of MilliQ water) were added [30]. The final supernatants were analyzed via spICP-MS (NexION 350 ICP-MS PerkinElmer, Waltham, MA, USA) to obtain the size distribution of *n*CeO_2_ present in roots and leaves.

### 2.5. Ce Concentration in Plant Fractions

Plant fractions were carefully washed with deionized water. The material was then oven-dried for three days at 60 °C. Subsequently, 0.3 g of dry plant fraction tissues were acid-digested on a CEM microwave oven (MARS Xpress, CEM, Matthews, NC, USA), using 9 mL of HNO_3_ (65%) and 1 mL of hydrogen peroxide (H_2_O_2_) in Teflon cylinders at 180 °C, according to the USEPA 3052 method [31]. After mineralization, plant extracts were filtered at room temperature under a fume hood with Whatman 0.45 μm PTFE membrane filters, and finally diluted and analyzed. Determinations of the total content of cerium were carried out using the NexION 350 ICP-MS. The accuracy of the analytical procedure adopted for ICP-MS analysis was checked by running standard solutions every 20 samples.

### 2.6. Data Analysis

Statistical analysis was carried out with one- and two-way ANOVA. A posteriori comparison of individual means was performed using Tukey’s test (*p* < 0.05). Before ANOVA, arcsine and logarithmic transformations were used to determine seed germination percentage and Ce concentration data, respectively. spICP-MS data on *n*CeO_2_ size distribution were processed using Syngistix Nano Application Module software and interpolated with Gaussian curves.

## 3. Results

### 3.1. Characterization of nCeO_2_

**Figure 2:**
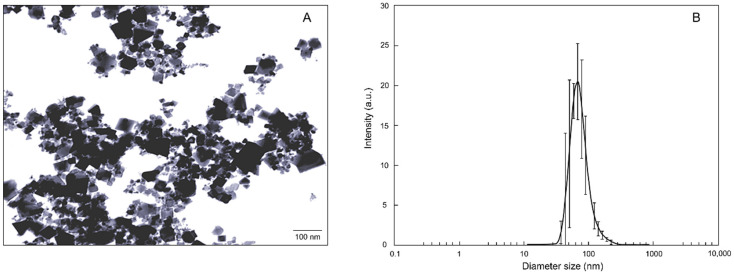
(**A**) Particle size distribution obtained by dynamic light scattering (DLS). (**B**) Transmission electron microscopy (TEM) image of *n*CeO_2_ 25 nm suspension.

*n*CeO_2_ characterization data are showed in Table S1, Supplementary Materials. The Hd distribution of both the materials is in agreement with the value provided by the supplier. The *n*CeO_2_ exhibited a monodisperse size particle distribution with relatively low PDI. The highest particle size was 62.0 nm (Figure 2A). The relative Z-averages were much larger than that value due to particle aggregation (Figure 2B).

### 3.2. nCeO_2_ in Plant Fractions

Before setting up the experiment on the entire vegetative cycle of *S. flos-cuculi*, some preliminary observations were carried out to demonstrate that *n*CeO_2_ was assimilated by the roots of plants and subsequently translocated to the upper plant parts. They were evidently necessary to set up the subsequent experiment illustrated in this paper. At first, a test was carried out to demonstrate the entry of *n*CeO_2_ within germinating seeds of *S. flos-cuculi* seeds [32]. Subsequently, under the same conditions as the main experiment, we wanted to verify that even in the presence of a complex matrix (that is, the potting soil compared to the very simple conditions of the germination test) the roots of *S. flos-cuculi* were able to take up the *n*CeO_2_.

**Figure 3:**
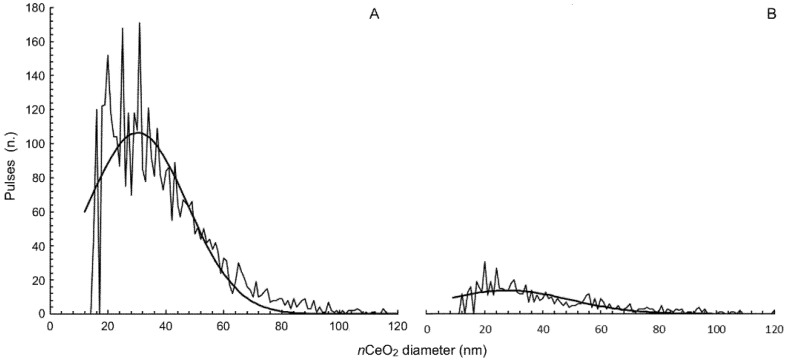
Particle size distribution of *n*CeO_2_ extracted after enzymatic digestion procedure from (**A**) roots and (**B**) leaves of *S. flos-cuculi*.

The results reported in Figure 3A,B clearly show that the *n*CeO_2_ was absorbed by the roots of *S. flos-cuculi*, and then moved upwards to reach the leaf tissues. The magnitude of pulses quantitatively represents the presence of *n*CeO_2_ in plant tissue; after the *n*CeO_2_ root absorption, only about 25% of nanoparticles moved to the plant leaves (Figure 3B). The mean size of *n*CeO_2_ was 33 ± 2 nm and 31 ± 1.5 nm in the roots and leaves, respectively (Table S2, Supplementary Materials), meaning that after being assimilated, *n*CeO_2_ did not undergo relevant aggregation. In the plant extract sample, the spICP-MS analysis also provided the concentration of the ionic form of an element dissolved from a nanostructure. In our case, the dissolved ionic Ce was very low, and equal to 4.86 ± 0.4 µg kg^−1^ in the roots and 0.08 ± 0.03 µg kg^−1^ in the leaves of *S. flos-cuculi*, respectively (Table S2, Supplementary Materials).

### 3.3. Plant Growth

An overall view of the experimental data relating to plant growth is showed in Table S3, Supplementary Materials, containing the results of a two–way ANOVA. In particular, the table reports the *p*-values testing the statistically significant effects of the *n*CeO_2_ dose (D1, D2 and D3), *n*CeO_2_ concentration (20 and 200 mg kg^−1^), and their interaction on biometric variables of *S. flos-cuculi*.

In broad terms, only the root dry weight (*p* = 0.0009 ***) responded in a statistically significant way to the dose of *n*CeO_2_, while this did not happen in the case of the other plant fractions and the leaf area. The factor “concentration” determined statistically significant effects in the case of root dry matter (*p* = 0.0281 *), stem dry matter (*p* = 0.0000 ***), and total plant dry weight (*p* = 0.0000 ***), as well. Finally, because the root apparatus was directly exposed to the soil matrix, as expected we recorded a statistically significant interaction of “dose X concentration” for root dry matter (*p* = 0.0018 *) (Table S3, Supplementary Materials).

**Figure 4:**
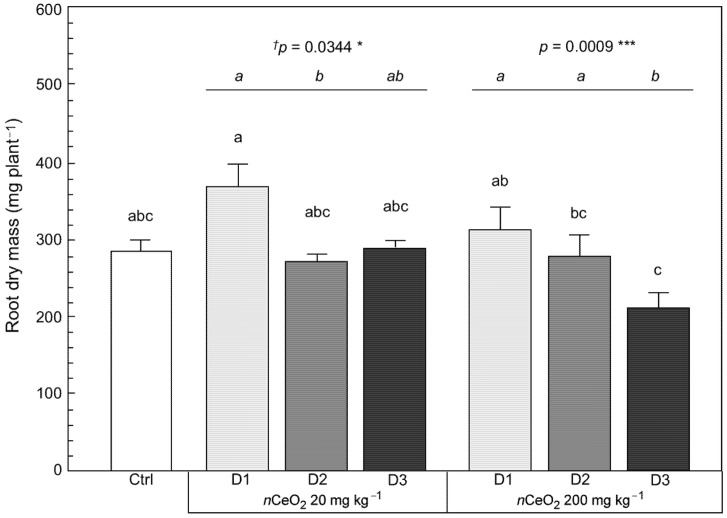
Root dry mass of *S. flos cuculi*. Comparison of effects based on single (D1) and repeated (D2, D3) applications of 20 and 200 mg kg^−1^ *n*CeO_2_, respectively. Letters indicate statistically significant difference between treatments (*p* ≤ 0.05) using one-way ANOVA followed by Tukey’s test. ^†^ One-way ANOVA *p*-value within each concentration: asterisks indicate the statistically significant difference of dose factor at * 0.05 ≥ *p* ≥ 0.01; ***p ≥ 0.001, respectively.

Carefully observing the effects of treatments on the vegetative development of *S. flos-cuculi*, some aspects of particular interest can be highlighted. As already mentioned, the root biomass dry weight, being the plant fraction directly exposed to the treatments, showed to be particularly sensitive to the experimental conditions. The development of the root apparatus responded positively to D1 (single dose of *n*CeO_2_ provided to pot soil before seed germination). At both concentrations of *n*CeO_2_, an increase of 29% (at 20 mg kg^−1^) and 9% (at 200 mg kg^−1^) in root biomass compared to the control was observed (Figure 4).

At the lowest concentration, the higher doses of *n*CeO_2_ (D2 and D3) did not promote the same effect detected for D1. The weight of the root biomass returned to a level very close to that of the control plants. This also occurred for D2 at the highest concentration (200 mg kg^−1^), while the response to D3 was a reduction of about 27% in root development compared to the control (Figure 4). Additionally, a statistical analysis was performed by isolating the concentration factor, i.e., testing the effect of single and repeated administration of *n*CeO_2_ to plants within the same concentration level. In this case, considering D1 as the reference within each concentration, we evaluated the effect of the additional doses of *n*CeO_2_ on the plant root biomass (Figure 4). Whether the single *n*CeO_2_ dose stimulated the production of root biomass, the second additional dose (D3), even though supplied to the plants at a late vegetative stage, resulted in a reduction in the root biomass. Compared to D1, we recorded a reduction in root dry matter of *S. flos-cuculi* by approximately 21% and 33%, for *n*CeO_2_ 20 mg kg^−1^ and 200 mg kg^−1^, respectively (Figure 4).

**Figure 5:**
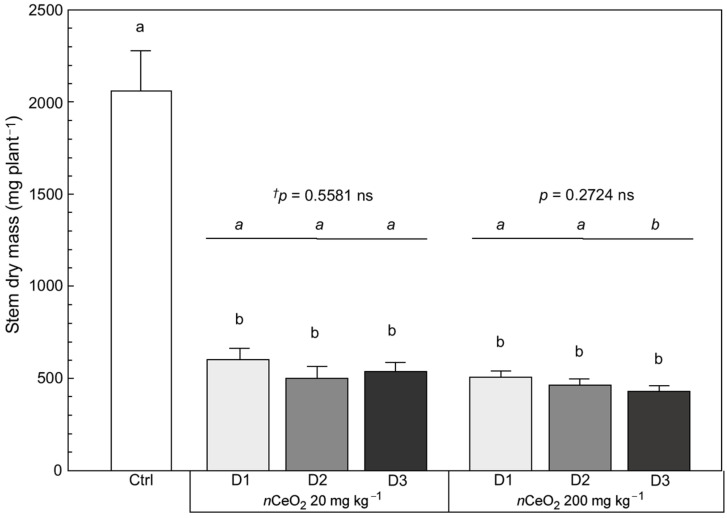
Stem dry mass of *S. flos cuculi*. Comparison of effects based on single (D1) and repeated applications (D2, D3) of 20 and 200 mg kg^−1^ *n*CeO_2_, respectively. Letters indicate statistically significant difference between treatments (*p* ≤ 0.05) using one-way ANOVA followed by Tukey’s test. ^†^ One-way ANOVA *p*-value within each concentration.

As reported in Table S3 Supplementary Materials, some other biometric variables were observed in plants. In particular, on the aboveground plant biomass, the number of stems and leaves for each plant were counted. The total leaf area per plant and the leaf dry matter were recorded as well. For these variables, the statistical analysis did not reveal significant effects of the treatments, whereas there was a very evident negative effect of *n*CeO_2_ on dry matter accumulation in the stems of *S. flos-cuculi* (Figure 5). Regardless of the concentration and dose of *n*CeO_2_, the negative effect of the treatment determined an average reduction of 75.5% in dry matter accumulation in the stems compared to the control.

As reported in Supplementary Materials (Figures S1–S4), the response to treatments of other biometric variables did not confirm either the stimulating effect highlighted on the case of root biomass or the negative effect on the dry matter accumulation on the stems *S. flos-cuculi*. Indeed, although the biomass of the stems was reduced, the architecture of the plants was not affected; the number of stems in the treated plants was no different from that of the control plants (Figure S1, Supplementary Materials). Even the number of leaves per plant, the leaf area per plant and the accumulation of dry matter in the leaves themselves were not affected by the treatments (Figures S2–S4, Supplementary Materials).

**Figure 6:**
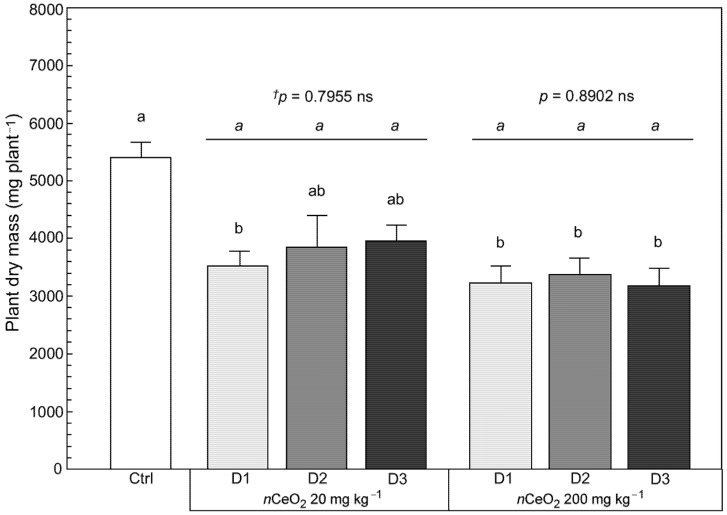
Plant dry mass of *S. flos cuculi*. Comparison of effects based on single (D1) and repeated applications (D2, D3) of 20 and 200 mg kg^−1^ *n*CeO_2_, respectively. Letters indicate statistically significant difference between treatments (*p* ≤ 0.05) using one-way ANOVA followed by Tukey’s test. ^†^ One-way ANOVA *p*-value within each concentration.

Figure 6 reports the plants’ total dry matter. Aggregating the different effects observed on the plant fractions could hide the impact of *n*CeO_2_ treatments. However, in our case this did not happen. Albeit to a lesser extent than that observed for the weight of the stems, the effect of *n*CeO_2_ on plant development is also visible on total biomass production. The negative effect of the treatments on the growth of *S. flos-cuculi* is statistically significant (*p* = 0.00000 ***), regardless of the *n*CeO_2_ dose and even at the lower concentration of nanoparticles (Figure 6).

After C fixation, the plant biomass was allocated according to species-specific patterns that are also influenced by environmental conditions as well as biotic and abiotic stress. Data regarding the dry weight of the plant fractions and the leaf area per plant were used to calculate new parameters (see Table S4, Supplementary Materials) that allowed us to evaluate the effects of *n*CeO_2_ treatments with a more accurate perspective. Additionally, in this case we can appreciate an overview of the effects of the experimental factors through the results of the two-way ANOVA (Table S5, Supplementary Materials). The effect of the “dose” factor was statistically significant only in the case of the root mass fraction (RMF) and the S/R ratio, while the response to the “concentration” factor was much more evident: only for specific leaf area (SLA) was the effect not statistically significant in the ANOVA. The interaction between the experimental factors was statistically significant for the RMF and the SLA (Table S5, Supplementary Materials). One-way ANOVA was used to evaluate the effects of treatments compared to the control and within the same concentration of *n*CeO_2_. 

Compared to the control and regardless of the *n*CeO_2_ concentration, the RMF was enhanced by D1, whereas D2 and D3 determined a subsequent drop of RMF. At the lowest concentration of *n*CeO_2_ concerning D1, we observed an almost-equal reduction in RMF in response to D2 and D3 (−33%). Additionally, at the highest concentration of *n*CeO_2_, the response to D2 and D3 was negative, although in this case it was gradual, with the reduction in RMF concerning D1 equal to −17% and −33%, for D2 and D3, respectively. However, the RMF of D2 and D3 treated plants was always higher than the control plants (Table 1).

SLA did not respond to the single experimental factors; however, ANOVA revealed a statistically significant effect for the interaction “dose X concentration” (*p* = 0.0243 *). Regarding the effects of the treatments, we observed a possible SLA stimulating effect of *n*CeO_2_ 20 mg kg^−1^ D1 and D2. At the same time, a certain variability prevented this empirical evidence from being statistically verified, whereas we observed a significant reduction in SLA in plants that received D3 compared to the controls (Table 1). In plants of *S. flos-cuculi* treated with *n*CeO_2_ 200 mg kg^−1^, SLA responded differently (*p* = 0.0243 *). Indeed, a slight reduction in SLA compared to the control due to treatment D1 (−4.7%) is associated with an evident increase in this parameter in response to treatments D2 and D3 (+10.7% and +18.6% greater than the control, respectively) (Table 1). Further ratios calculated from biometric variables (Stem mass fraction SMF, Leaf mass fraction LMF, Shoot to root ratio Shoot/Root and Leaf area ratio LAR) are reported in Supplementary Materials (Figures S5–S8).

### 3.4. Cerium Concentration in Plant Fractions

A general view of the Ce uptake and accumulation in plant tissues as affected by experimental factors is given in Table S6, Supplementary Materials. The factor “dose” result was statistically significant only for Ce concentration in plant stems (*p* = 0.0000 ***), while the Ce accumulation in each plant tissue, as expected, increased responding to the factor “concentration”. A statistically significant interaction “dose x concentration” was observed in the roots (*p* = 0.0313 *) and stems (*p* = 0.0021 **).

Table 2 reports data regarding the Ce concentrations in plant fractions. At first glance, the data indicate that plant Ce uptake was not very high compared to the treatments. Concerning the plant fractions, as expected, Ce in the roots was higher than the others.

On average, the treatment concentration of Ce in the root tissues (3074 µg kg^−1^) was four times higher than that of the stems (779 µg kg^−1^) and two times higher than that found in the leaves (1594 µg kg^−1^), respectively. However, we do not observe a clear and statistically significant response to the *n*CeO_2_ doses regardless of the treatment concentration. However, the statistically significant interaction “dose X concentration” is explained by the different response in terms of Ce accumulation in roots after dose D3 *n*CeO_2_ 200 mg kg^−1^ that was about 49% higher than the average D1–D2. (Table 2).

After being taken up by the roots, a fraction of Ce moved towards the aerial plant fractions to be allocated in the stems. A statistically significant effect of the dose factor and of the interaction is visible by observing the concentration of Ce in the stems (Table 2). Here, although the highest Ce concentration was detected at D3 *n*CeO_2_ 20 mg kg^−1^, the most evident effect of the “dose” factor can be appreciated for plants exposed to *n*CeO_2_ 200 mg kg^−1^ (*p* = 0.0015 **; Table 2). Finally, the leaves represent the final allocation of Ce in plant aerial biomass. Here, Ce accumulation was higher in than in stems; however, due to a certain variability, it was not possible to statistically verify a significant effect of the experimental treatments (Table 2)

## 4. Discussion

Only in 2012 were the effects of ENMs over the whole plant cycle studied [33]. In soybeans (*Glycine max* L.), it was demonstrated that Ce concentrations in the roots and the concentration of *n*CeO_2_ in soil were correlated. Nanoceria negatively influenced the yield of soybean and N_2_-fixation by affecting the efficiency of the symbiotic system established with *Bradyrhizobium*: a dramatic example of the influence on cultivated plants and wild species’ ecological services, as well.

A large body of literature reports negative responses observed at different plant growth stages. When germinating seeds are exposed to *n*CeO_2_, other effects could be verified, basically depending on particle size and concentration. Additionally, statistically significant species-specific responses were reported, regarding root elongation being more sensitive to *n*CeO_2_ than germination [34,35,36]. Other studies explored the physiological implications of the *n*CeO_2_ plant uptake, concluding that plants responded to the treatments increasing the antioxidant enzyme activities. However, the oxidative stress induced by high concentrations of *n*CeO_2_ cannot be attenuated by the antioxidant system [37,38,39,40].

The growth of *S. flos-cuculi* was negatively affected by *n*CeO_2_. Suppose the root apparatus development in plants treated at the lowest *n*CeO_2_ concentration has not undergone apparent alterations at the highest concentration; in this case, the effect is evident and progressively increases as the *n*CeO_2_ dose increases. The impact of *n*CeO_2_ on plant growth was much more apparent in the biomass of plant stems. We observed a slowdown in plant growth. The number of plant stems did not change, but they were shorter than the controls’. No statistically significant evidence was found regarding the effects of treatments on leaf biomass (evaluated by counting the number of leaves per plant, the leaf area, and the leaf dry weight). However, likely the relevant data variability detected in the treated plants compared to that of the control plants was an early signal of plant stress.

SLA is a very informative parameter in plant ecology. The total leaf area ratio to total leaf dry mass correlates with whole-plant growth linking C gain and water loss [41]. Even though we calculated the SLA using data from a single biomass sampling at the end of the growth cycle of *S. flos-cuculi*, the response of SLA to the *n*CeO_2_ treatments allowed us to interpret the experimental data more effectively. In particular, the increase in SLA responded to the dose of *n*CeO_2_ received by the plant. Moreover, this could be a consequence of the slowing of the vegetative growth rate and could lead us to conclude that the *n*CeO_2_ negatively affects the C accumulation by leaf tissues. Our data do not allow us to identify the specific cause precisely. However, this observation corroborates the literature evidence regarding the slowing of the plant growth cycle [42] and photosynthesis, both in terrestrials and aquatic plants [43,44].

The growing number of nanotechnology applications in various fields inevitably results in the release of nanomaterials into the environment. Models demonstrated that wastewater and sewage sludge are the primary vectors by which ENMs end up in the environment [45]. Apart from the quantitative aspect, nanomaterials’ flows can occur differently concerning the position of the target to the source (e.g., a single massive event or events repeated over time). Literature papers concerning the effects of ENMs on plants always report experiments where the nanomaterials were applied in a single concentration, whereas a more realistic exposure scenario involves repeated pulses.

In our study, plants of *S. flos-cuculi* were grown in soil amended with *n*CeO_2_. The experimental design was conceived assuming that the soil could receive different *n*CeO_2_ pulses over time, thereby obtaining three different doses of *n*CeO_2_ supplied at different growth stages of *S. flos-cuculi*. At the moment, we cannot compare our data with other works having the same experimental approach. We have already cited a paper reporting Ag and Cu nanoparticles’ effects on seedlings of *Pinus sylvestris* and *Quercus robur*. A single dose of nanomaterials was administered to plants by three subsequent foliar applications in that study, whereas in our experiment were provided three amounts of *n*CeO_2_. However, in both experiments, the experimental factor “dose” or merely the phenological stage at which plants received the treatments showed some influence on the consequences of the treatment. Therefore, this early indication suggests that this type of study should be further developed. Other studies of soil ecology have used the same approach. In particular, it was demonstrated that soil enzyme activity is differently affected by repeated ENM doses, indicating that additive effects occur [46,47]. It will be necessary to compile these different works to achieve a complete evaluation of the effects of ENMs on the soil–plant system.

## Figures and Tables

**Table 1:** Root mass fraction (RMF), shoot to root ratio (S/R ratio), and specific leaf area (SLA) ± standard deviation of *S. flos-cuculi* grown in presence of different inputs of 20–200 mg kg^−1^ *n*CeO_2_. Statistically significant differences (*p* ≤ 0.05) are indicated by the letters using one-way ANOVA followed by Tukey’s test. Dashed box indicate ANOVA *p*-values (*p* ≤ 0.05) within the *n*CeO_2_ concentration. ns: not significant at *p* ≤ 0.05; * and ** significant at *p* ≤ 0.05 and *p* ≤ 0.01.

Treatment	Dose	RMF	SLA
		(g g^−1^)	(m^2^ kg^−1^)
Ctrl	D0	0.054 ± 0.003 c	25.3 ± 0.70 ab
*n*CeO_2_ 20 mg kg^−1^	D1	0.117 ± 0.009 a	25.7 ± 1.21 ab
	D2	0.078 ± 0.008 bc	27.3 ± 1.32 ab
	D3	0.077 ± 0.007 bc	24.4 ± 1.42 b
		*p* = 0.0028 **	*p* = 0.2801 ns
*n*CeO_2_ 200 mg kg^−1^	D1	0.105 ± 0.009 ab	24.1 ± 1.31 b
	D2	0.087 ± 0.01 abc	28 ± 1.63 ab
	D3	0.070 ± 0.006 c	30 ± 1.36 a
		*p* = 0.0271 *	*p* = 0.0243 *

**Table 2:** Ce concentration in plant fraction and Ce translocation factor in *S. flos cuculi* grown in the presence of different inputs of *n*CeO_2_ (20 and 200 mg kg^−1^.) Data are mean *±* standard deviation. Statistically significant differences (*p* ≤ 0.05) are indicated by the letters using one-way ANOVA follow by Tukey’s test. Dashed boxes indicate ANOVA *p*-values (*p* ≤ 0.05) within *n*CeO_2_ concentration. ns: not significant at *p* ≤ 0.05; ** significant at *p* ≤ 0.01.

Treatment	Dose	Ce roots	Ce stems	Ce leaves
		(µg kg^−1^)	(µg kg^−1^)	(µg kg^−1^)
Ctrl	D0	546 ± 390 b	154 ± 125 b	254 ± 198 b
*n*CeO_2_ 20 mg kg^−1^	D1	1300 ± 112 b	333 ± 281 b	1083 ± 70 ab
	D2	2407 ± 793 b	477 ± 172 ab	1240 ± 170 ab
	D3	2670 ± 1130 b	1450 ± 918 a	1770 ± 96.7 a
		*p* = 0.1653 ns	*p* = 0.0988 ns	*p* = 0.3638 ns
*n*CeO_2_ 200 mg kg^−1^	D1	3023 ± 700 ab	573 ± 87 ab	1580 ± 60.7 ab
	D2	3130 ± 2210 ab	816 ± 91 ab	2063 ± 41.8 a
	D3	5910 ± 1140 a	1023 ± 61 ab	1827 ± 24 a
		*p* = 0.0941 ns	*p* = 0.0015 **	*p* = 0.4643 ns

## Data Availability

The data presented in this study are available on request from the corresponding author.

## Supplementary Materials

The following are available online at https://www.mdpi.com/2079-4991/11/1/229/s1: Figure S1. Number of stems per plant in specimens of *S. flos-cuculi*. Comparison of effects based on single (D1) and repeated applications (D2, D3) of 20 and 200 mg kg^−1^ *n*CeO_2_, respectively. Letters indicate statistically significant difference between treatments (*p* ≤ 0.05) using one-way ANOVA followed by Tukey’s test. ^†^ One-way ANOVA *p*-value within each concentration. Figure S2. Number of leaves per plant in specimens of *S. flos-cuculi*. Comparison of effects based on single (D1) and repeated applications (D2, D3) of 20 and 200 mg kg^−1^ *n*CeO_2_, respectively. Letters indicate statistically significant difference between treatments (*p* ≤ 0.05) using one-way ANOVA followed by Tukey’s test. ^†^ One-way ANOVA *p*-value within each concentration. Figure S3. Total leaf area in plants of *S. flos-cuculi*. Comparison of effects based on single (D1) and repeated applications (D2, D3) of 20 and 200 mg kg^−1^ *n*CeO_2_, respectively. Letters indicate statistically significant difference between treatments (*p* ≤ 0.05) using one-way ANOVA followed by Tukey’s test. ^†^ One-way ANOVA *p*-value within each concentration. Figure S4. Leaf dry matter in plants of *S. flos-cuculi*. Comparison of effects based on single (D1) and repeated applications (D2, D3) of 20 and 200 mg kg^−1^ *n*CeO_2_, respectively. Letters indicate statistically significant difference between treatments (*p* ≤ 0.05) using one-way ANOVA followed by Tukey’s test. ^†^ One-way ANOVA *p*-value within each concentration. Figure S5. Stem mass fraction of *S. flos-cuculi*. Comparison of effects based on single (D1) and repeated applications (D2, D3) of 20 and 200 mg kg^−1^ *n*CeO_2_, respectively. Letters indicate statistically significant difference between treatments (*p* ≤ 0.05) using one-way ANOVA followed by Tukey’s test. ^†^ One-way ANOVA *p*-value within each concentration. Figure S6. Leaf mass fraction of *S. flos-cuculi*. Comparison of effects based on single (D1) and repeated applications (D2, D3) of 20 and 200 mg kg^−1^ *n*CeO_2_, respectively. Letters indicate statistically significant difference between treatments (*p* ≤ 0.05) using one-way ANOVA followed by Tukey’s test. ^†^ One-way ANOVA *p*-value within each concentration. Figure S7. Shoot to root ratio in *S. flos-cuculi*. Comparison of effects based on single (D1) and repeated applications (D2, D3) of 20 and 200 mg kg^−1^ *n*CeO_2_, respectively. Letters indicate statistically significant difference between treatments (*p* ≤ 0.05) using one-way ANOVA followed by Tukey’s test. ^†^ One-way ANOVA *p*-value within each concentration. Figure S8. Leaf area ratio of *S. flos-cuculi*. Comparison of effects based on single (D1) and repeated applications (D2, D3) of 20 and 200 mg kg^−1^ *n*CeO_2_, respectively. Letters indicate statistically significant difference between treatments (*p* ≤ 0.05) using one-way ANOVA followed by Tukey’s test. ^†^ One-way ANOVA *p*-value within each concentration. Table S1. Average, PDI and ζ-potentials of *n*CeO_2_ 25 nm. Table S2. Most frequent particle size, mean particle size, number of pulses and concentration of dissolved Ce determined by sp-ICP-MS analysis after enzymatic extraction from roots and leaves of Silene flos-cuculi. Table S3. Two-way ANOVA *p*-values testing the statistically significant effects of dose and concentration and their interaction of *n*CeO_2_ on biometric variables of *S. flos-cuculi*. Table S4. Biomass allocation variables calculated from plant measurements (Poorter et al., 2011). Table S5. Two-way ANOVA *p*-values testing the statistically significant effects of dose and concentration and their interaction of *n*CeO_2_ on growth indices of *S. flos-cuculi*. Table S6. Two-way ANOVA *p*-values testing the statistically significant effects of dose and concentration and their interaction on Ce concentrations in fractions of *S. flos-cuculi* and Ce translocation factor.
