# Supplementary material for: Single and Repeated Applications of Cerium Oxide Nanoparticles Differently Affect the Growth and Biomass Accumulation of Silene flos-cuculi L. (Caryophyllaceae)
Source: Nanomaterials (Basel). 2021 Jan 16;11(1):229. doi: 10.3390/nano11010229 (PMC7829812; doi:10.3390/nano11010229)
Supplement: Supplementary file 1 [file nanomaterials-11-00229-s001.pdf]

Supporting Information

Single and Repeated Applications of Cerium Oxide Nanoparticles Differently Affect the Growth and Biomass Accumulation of *Silene flos-cuculi L.* (*Caryophyllaceae*)

Daniel Lizzi [1,2], Alessandro Mattiello [2], Barbara Piani [1], Emanuele Gava [3], Guido Fellet [1] and Luca Marchiol [1,*]

[1] Department of AgriFood, Environmental and Animal Science, University of Udine, via delle Scienze 206, 33100 Udine, Italy; lizzi.daniel.1@spes.uniud.it (D.L.); alessandro.mattiello@uniud.it; barbara.piani@uniud.it (B.P.); guido.fellet@uniud.it (G.F.)

[2] Department of Life Sciences, University of Trieste, Via Licio Giorgieri 10, 34127, Trieste, Italy;

[3] Laboratory of Inorganic Micro-Pollutants Regional Environmental Protection Agency of Friuli Venezia Giulia (ARPA-FVG), Via Colugna 42, 33100 Udine, Italy; emanuele.gava@arpa.fvg.it

[*] Correspondence: marchiol@uniud.it

Results

Nanoparticles characterization

Table S1. Average, PDI and ζ - potentials of nCeO2 25 nm.

Material	Z - average (nm)	PDI	ζ - potential (mV)
nCeO_2 25 nm	126.7 ± 1.0	0.17 ± 0.01	39.2 ± 1.1

nCeO_2 *in plant fractions*

Table S2. Most frequent particle size, mean particle size, number of pulses and concentration of dissolved Ce determined by sp–ICP–MS analysis after enzymatic extraction from roots and leaves of *Silene flos-cuculi*.

Plant fraction	Replicate	*n*CeO_2 most frequent size (nm)	*n*CeO_2 mean size (nm)	Pulses (n)	Dissolved Ce (μg kg^{-1})
Roots	1	31	35	3279	5.32
Roots	2	27	31	2136	4.58
Roots	3	30	33	2091	4.69
Mean±†SD		29 ± 2.08†	33 ± 2	2502 ± 673	4.86 ± 0.40
Leaves	1	25	31	630	0.05
Leaves	2	26	29	653	0.08
Leaves	3	28	32	583	0.10
Mean±SD		26 ± 1.53	31 ± 1.53	622 ± 35.7	0.08 ± 0.03

† Standard Deviation

Plant growth

Table S3. Two-way ANOVA p values testing the statistically significant effects of dose and concentration and their interaction of *n*CeO_2 on biometric variables of *Silene flos-cuculi*. ns: not significant at $p \leq 0.05$; * p≤ 0.05, *** p≤ 0.001.

Variable	Dose	Concentration	Dose x Concentration
Roots DW	0.0009 ***	0.0281 *	0.0179 *
Stems per plant	0.0942 ns	0.4020 ns	0.5681 ns
Stems DW	0.8874 ns	0.0000 ***	0.9956 ns
Leaves per plant	0.2645 ns	0.7375 ns	0.7963 ns
Leaf area	0.7498 ns	0.7028 ns	0.8419 ns
Leaves DW	0.8990 ns	0.6052 ns	0.9704 ns
Total DW	0.8436 ns	0.0000 ***	0.9885 ns

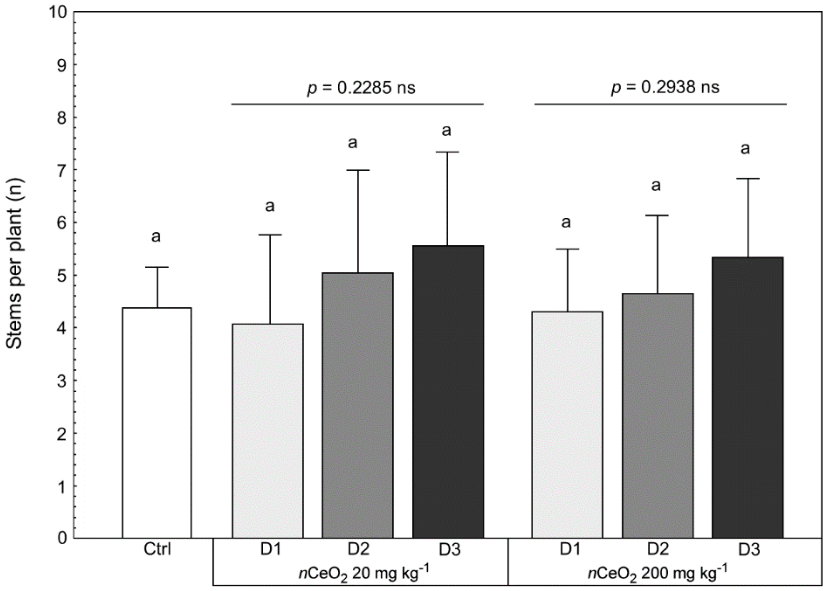

Figure S1. Number of stems per plant in specimens of *S. flos cuculi*. Comparison of effects based on single (D1) and repeated applications (D2, D3) of respectively 20 and 200 mg kg^{-1} *n*CeO_2. Letters indicate statistically significant difference between treatments ($p \leq 0.05$) using one-way ANOVA followed by Tukey's test. †One-way ANOVA p value within each concentration.

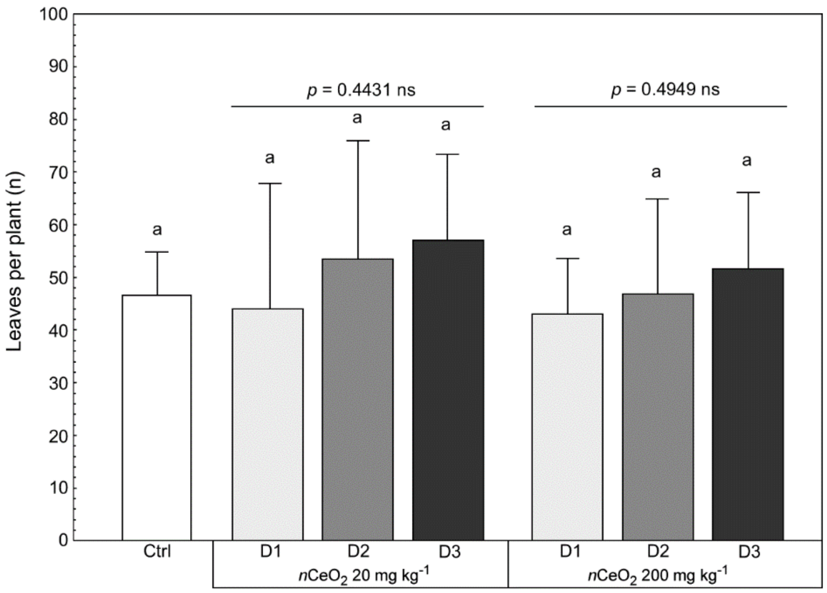

Figure S2. Number of leaves per plant in specimens of *S. flos cuculi*. Comparison of effects based on single (D1) and repeated applications (D2, D3) of respectively 20 and 200 mg kg^{-1} *n*CeO_2. Letters indicate statistically significant difference between treatments ($p \leq 0.05$) using one-way ANOVA followed by Tukey's test. †One-way ANOVA p value within each concentration.

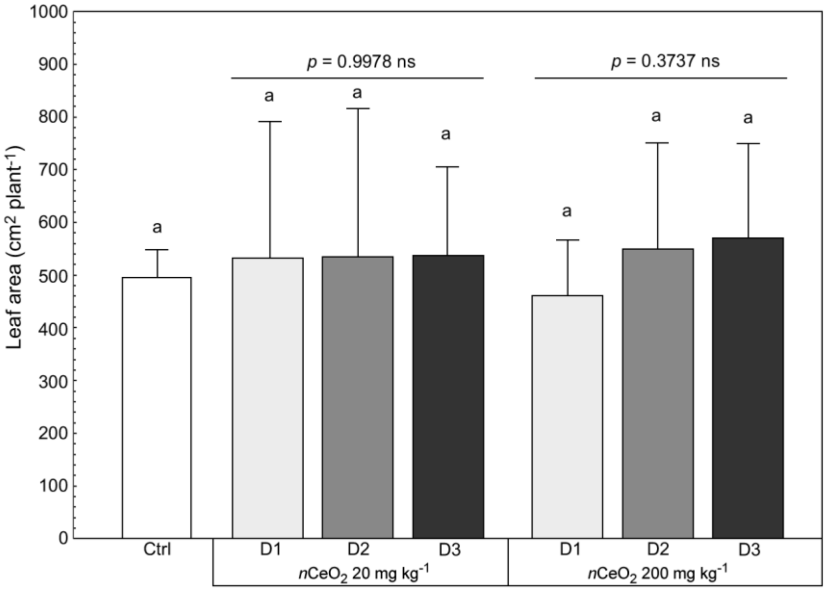

Figure S3. Total leaf area in plants of *S. flos cuculi*. Comparison of effects based on single (D1) and repeated applications (D2, D3) of respectively 20 and 200 mg kg^{-1} *n*CeO_2. Letters indicate statistically significant difference between treatments ($p \leq 0.05$) using one-way ANOVA followed by Tukey's test. †One-way ANOVA p value within each concentration.

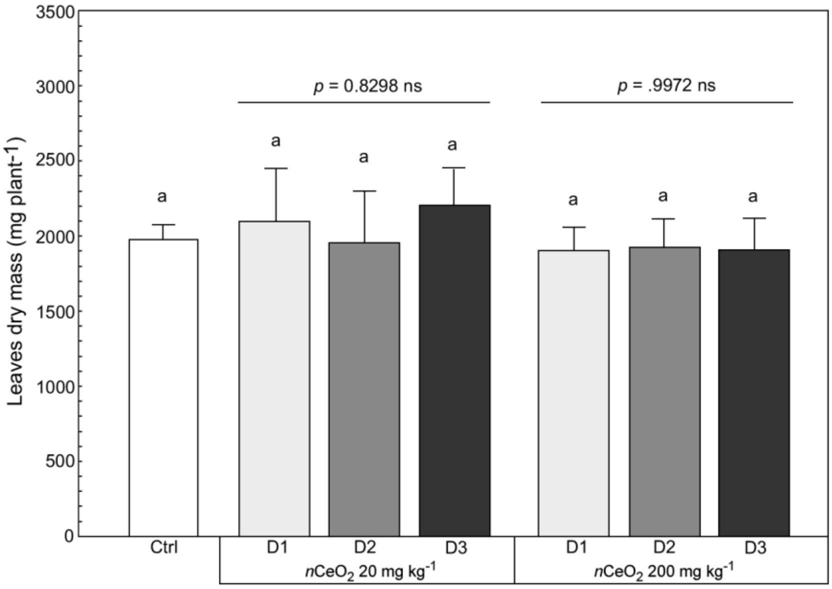

Figure S4. Leaves dry matter in plants of *S. flos cuculi*. Comparison of effects based on single (D1) and repeated applications (D2, D3) of respectively 20 and 200 mg kg^{-1} *n*CeO_2. Letters indicate statistically significant difference between treatments ($p \leq 0.05$) using one-way ANOVA followed by Tukey's test. [†]One-way ANOVA p value within each concentration.

Table S4. Biomass allocation variables calculated from plant measurements (Poorter et al, 2011).

Variable	Abbreviation	Definition	Unit
Root Mass Fraction	RMF	Root dry mass/Total plant dry mass	g g^{-1}
Stem Mass Fraction	SMF	Stem dry mass/Total plant dry mass	g g^{-1}
Leaf Mass Fraction	LMF	Leaf dry mass/Total plant dry mass	g g^{-1}
Shoot to Root ratio	S/R ratio	(Leaf + Stem dry mass)/Root dry mass	g g^{-1}
Leaf Area Ratio	LAR	Leaf area/Total plant dry mass	m^2 kg^{-1}
Specific Leaf Area	SLA	Leaf area/Leaf dry mass	m^2 kg^{-1}

Table S5. Two-way ANOVA p values testing the statistically significant effects of dose and concentration and their interaction of *n*CeO_2 on growth indices of *Silene flos-cuculi* ns: not significant at $p \leq 0.05$; * p≤ 0.05, ** $p \leq 0.01$ and *** p≤ 0.001.

Variable	Dose	Concentration	Dose x Concentration
RMF	0.0001 ***	0.0001 ***	0.0201 *
SMF	0.3103 ns	0.0000 ***	0.8223 ns
LMF	0.7438 ns	0.0000 ***	0.9545 ns
S/R ratio	0.0011 **	0.0000 ***	0.0593 ns
LAR	0.7703 ns	0.0000 ***	0.3778 ns
SLA	0.1438 ns	0.0958 ns	0.0251 *

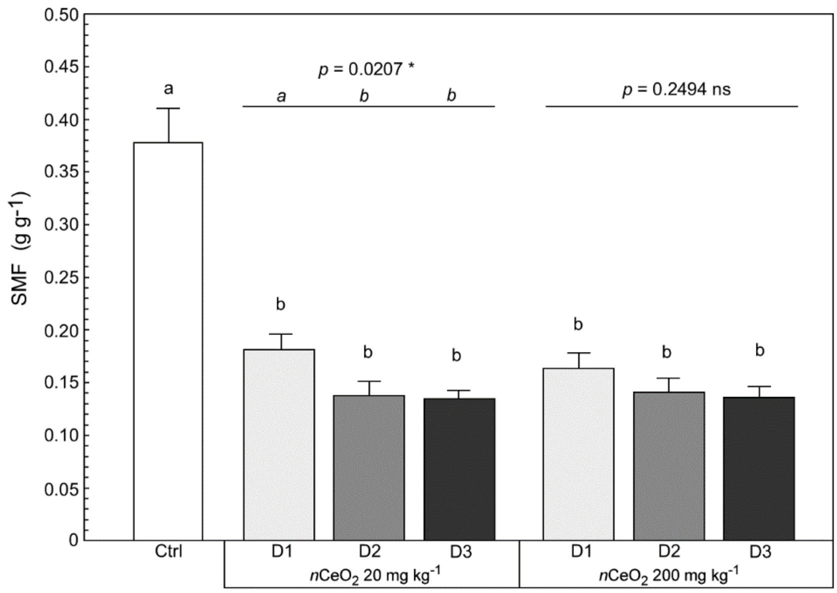

Figure S5. Stem mass fraction of *S. flos cuculi*. Comparison of effects based on single (D1) and repeated applications (D2, D3) of respectively 20 and 200 mg kg^{-1} nCeO$_2$. Letters indicate statistically significant difference between treatments ($p \leq 0.05$) using one-way ANOVA followed by Tukey's test. †One-way ANOVA p value within each concentration.

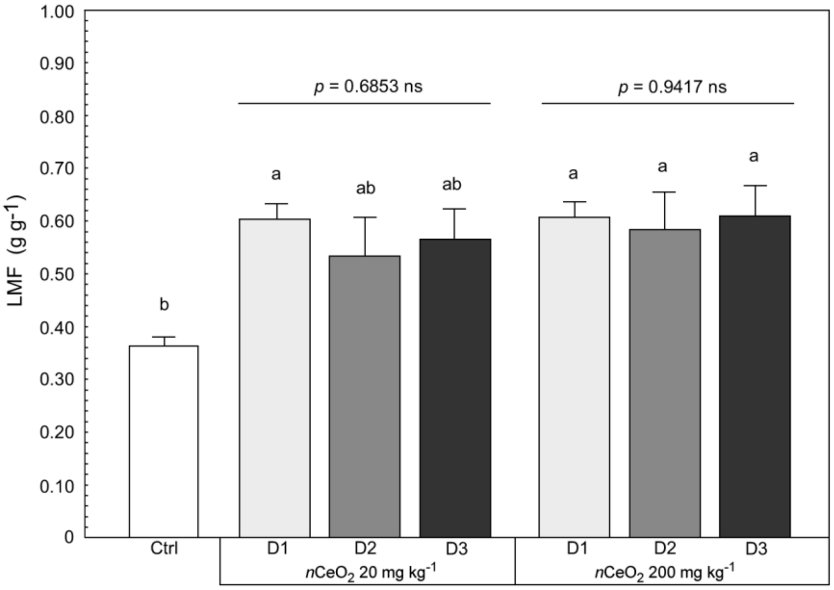

Figure S6. Leaf mass fraction of *S. flos cuculi*. Comparison of effects based on single (D1) and repeated applications (D2, D3) of respectively 20 and 200 mg kg^{-1} nCeO$_2$. Letters indicate statistically significant difference between treatments ($p \leq 0.05$) using one-way ANOVA followed by Tukey's test. †One-way ANOVA p value within each concentration.

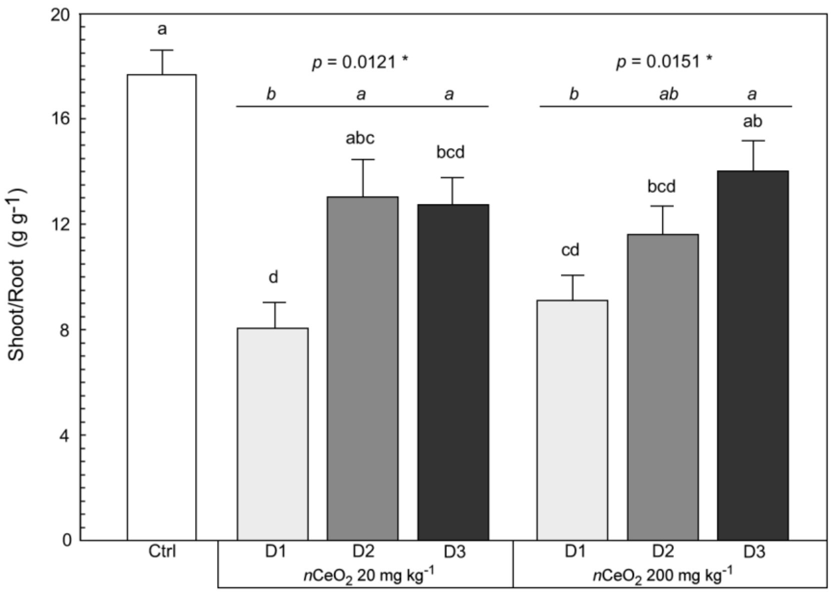

Figure S7. Shoot to root ratio in *S. flos cuculi*. Comparison of effects based on single (D1) and repeated applications (D2, D3) of respectively 20 and 200 mg kg^{-1} *n*CeO_2. Letters indicate statistically significant difference between treatments ($p \leq 0.05$) using one-way ANOVA followed by Tukey's test. †One-way ANOVA p value within each concentration.

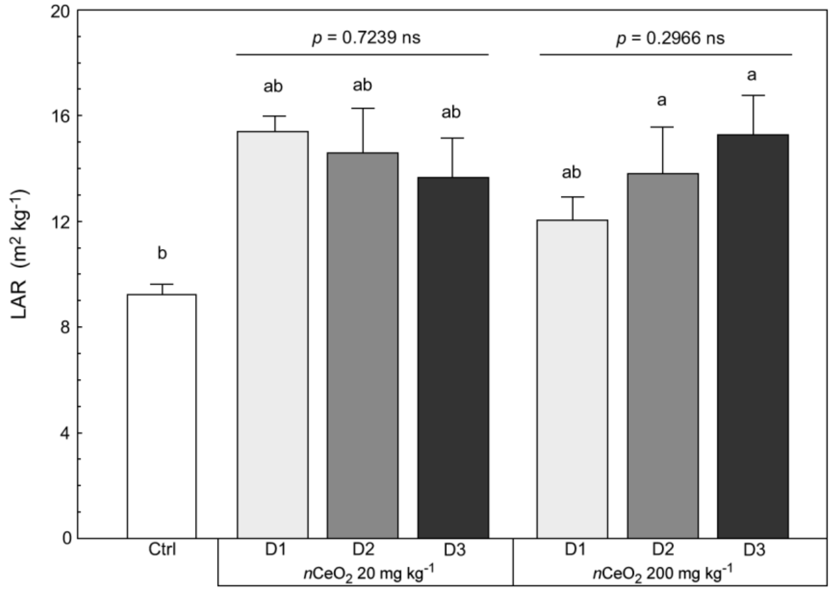

Figure S8. Leaf area ratio of *S. flos cuculi*. Comparison of effects based on single (D1) and repeated applications (D2, D3) of respectively 20 and 200 mg kg^{-1} *n*CeO_2. Letters indicate statistically significant difference between treatments ($p \leq 0.05$) using one-way ANOVA followed by Tukey's test. †One-way ANOVA p value within each concentration.

Ce accumulation plant fractions

Table S6. Two-way ANOVA p values testing the statistically significant effects of dose and concentration and their interaction on Ce concentration in fractions of *S. flos-cuculi* and Ce translocation factor. ns: not significant at $p \leq 0.05$; * p≤ 0.05, ** p ≤ 0.01 and *** p≤ 0.001.

Variable	Dose	Concentration	Dose x Concentration
Ce roots	0.0811 ns	0.0000 ***	0.0313 *
Ce stems	0.0000 ***	0.0000 ***	0.0021 **
Ce leaves	0.8188 ns	0.0005 ***	0.0786 ns
$TF_{root\text{-}leaves}$	0.0395 *	0.3402 ns	0.3775 ns
